# Alfalfa Polysaccharide Improves Rabbit Growth by Modulating Gut Microbiota and Suppressing Inflammation Through PPARγ/NF-κB Pathway

**DOI:** 10.3390/ijms27020994

**Published:** 2026-01-19

**Authors:** Junying Xu, Fang Li, Yuanxin Zhu, Dongmei Liu, Wenjing Duan, Ran Yu, Boshuai Liu, Hao Sun, Zhichang Wang, Defeng Li, Xiaoyan Zhu, Yinghua Shi, Yalei Cui

**Affiliations:** 1 College of Animal Science and Technology, Henan Agricultural University, No. 15 Longzihu University Area, Zhengdong New District, Zhengzhou 450046, China; ying0508323@163.com (J.X.); lf2468365563@163.com (F.L.); 17525905289@163.com (Y.Z.); 13271437098@163.com (D.L.); duanwenjing0620@163.com (W.D.); sgqt2023221yr@163.com (R.Y.); boshuailiu@126.com (B.L.); sunhao@henau.edu.cn (H.S.); zcwang@henau.edu.cn (Z.W.); leadephone@126.com (D.L.); zxy_0512@163.com (X.Z.); annysyh@henau.edu.cn (Y.S.); 2 Henan Key Laboratory of Innovation and Utilization of Grassland Resources, Zhengzhou 450002, China; 3 Henan Forage Engineering Technology Research Center, Zhengzhou 450002, China

**Keywords:** alfalfa polysaccharide, rabbit, growth performance, meat quality, gut microbiota, inflammation, butyrate

## Abstract

Meat rabbits are ideal meat-producing animals. However, weaning-induced intestinal inflammation often leads to growth delays, and severe cases impair breeding efficiency. Alfalfa polysaccharides (APSs) have antioxidant and anti-inflammatory properties, making them potential natural alternatives to antibiotics. To date, relatively limited research has been conducted on APS in meat rabbits. This research investigated the effects of APS on growth performance, intestinal inflammation, and meat quality in rabbits. Eighty healthy rabbits were randomized into four treatment groups, each group consisting of five replicates, with four rabbits per replicate. The four experimental groups were the control group (CON, basal diet), 400 mg/kg APS group (basal diet + 400 mg/kg APS), 800 mg/kg APS group (basal diet + 800 mg/kg APS), and 1200 mg/kg APS group (basal diet + 1200 mg/kg APS). The results indicated that adding 800 mg/kg APS to the diet significantly increased ADG (*p* < 0.001) and reduced F/G (*p* = 0.008). With increasing APS supplementation levels, slaughter weight (*p* = 0.035), eviscerated weight (*p* = 0.020), semi-eviscerated weight (*p* = 0.015), and semi-eviscerated yield percentage (*p* = 0.035) were all significantly increased. Additionally, dripping loss in muscle was significantly reduced in the 800 mg/kg APS group (*p* = 0.006). In addition, the villus height of the small intestine and the expression of tight junctions were significantly increased by 800 mg/kg APS supplementation, which reduced intestinal permeability and lowered levels of intestinal inflammatory mediators by inhibiting the PPARγ/NF-κB pathway. Additionally, a diet with APS significantly increased the abundance of *Flavonifractor*, a butyrate-producing bacterium in the cecum. Cell assays further demonstrated that butyrate could inhibit the release of inflammatory cytokines from RAW264.7 via the PPARγ/NF-κB pathway. In conclusion, APS improved growth performance by reshaping the gut microbiota and increasing the level of butyrate in the cecum, further inhibiting intestinal inflammation through the PPARγ/NF-κB signaling pathway.

## 1. Introduction

Rabbits, characterized by a life cycle with short gestation periods along with high production efficiency, are ideal animals for meat production [1]. However, weaning-induced intestinal inflammatory diseases leading to growth delays or even death severely limit production efficiency in commercial meat rabbit farming [2]. The misuse of antibiotics in animal production and disease prevention has long been opposed. In recent years, plant extracts extracted through appropriate processing have gradually become a research focus as alternatives to antibiotics in livestock, owing to their diverse functionalities, lack of drug resistance, and non-residual properties [3].

Plant bioactive compounds extracted through modern processing technologies—such as flavonoids, saponins, and polysaccharides—have been demonstrated to promote animal growth and maintain gut health in integrated studies employing multiple omics technologies, including transcriptomics, proteomics, and network pharmacology approaches [4,5]. Alfalfa polysaccharide (APS), as a natural, safe, and highly effective plant polysaccharide with multiple biological activities, is a highly desirable feed additive. Research has shown that APS promotes immune cell proliferation and promotes immunoglobulin production, thereby enhancing the immunity of the body [6]. Under disease conditions, oxidative stress in animals generates excessive free radicals that damage cell membranes, posing a potential threat to health. APS can play an antioxidant role by scavenging free radicals, protecting cells from oxidative stress, and thereby alleviating inflammation within the body [7,8]. Multiple studies have shown that APS regulates gut microbiota structure in animals, increasing probiotic abundance and suppressing pathogenic bacteria [9,10]. In summary, APS has the potential to be used as natural antibiotic alternatives in animal production due to their diverse biological activities. Research on APS has mainly focused on pigs, chickens, and ruminants, with limited studies about rabbits. APS offers promising benefits in terms of gut health, meat quality, and farming efficiency in weaned rabbits. This makes it an effective solution for sustainable animal husbandry.

## 2. Result

### 2.1. Effects of APS on Growth, Slaughter Performance, and Meat Quality in Rabbit

**Figure 1:**
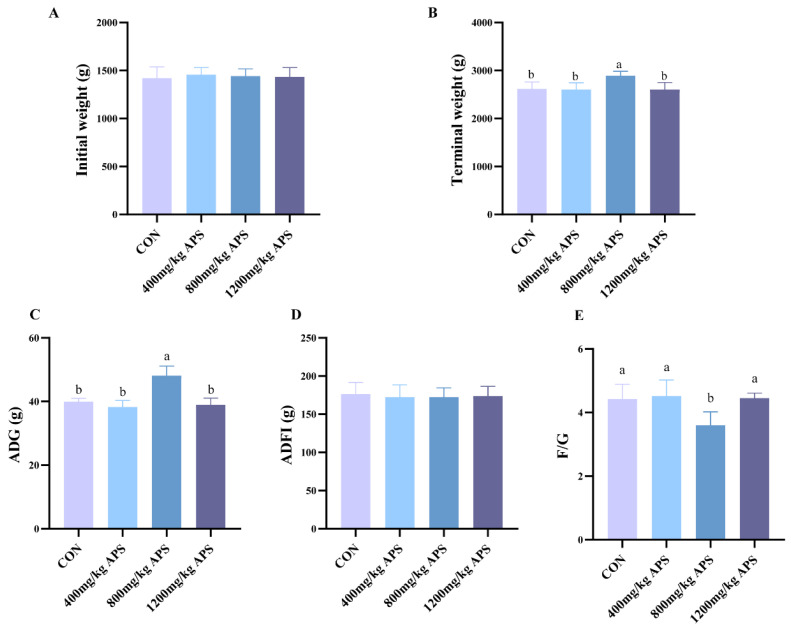
Effects of dietary supplementation with different dosages of APS on rabbit growth performance. (**A**) Initial weight of rabbit. (**B**) Terminal weight of rabbit. (**C**) Effects of APS on ADG of rabbit. (**D**) ADFI of rabbit. (**E**) The value of F/G. Data are presented as mean ± SD. Different letters indicate significant difference.

In the field of animal science and production research, growth performance and slaughter performance are the most direct and main indicators for evaluating the production efficiency of economic animals. As for growth performance (Figure 1), APS at a dosage of 800 mg/kg significantly increased the ADG and decreased F/G. No significant differences were observed in ADFI between the different groups.

With increasing dosage of APS, the slaughter weight, eviscerated weight, semi-eviscerated weight, eviscerated yield percentage, and semi-eviscerated yield percentage of rabbits were all increased (Table 1). Compared with CON, APS significantly increased the slaughter weight at the dosage of 1200 mg/kg (Table 1). As for meat quality (Table 1), the drip loss of meat was significantly reduced by APS at a dosage of 800 mg/kg. A comprehensive analysis of growth performance, slaughter performance, and meat quality showed that the optimal application effect can be achieved with an APS additive dosage of 800 mg/kg.

### 2.2. Effect of APS on the Intestinal Barrier in Rabbit

**Figure 2:**
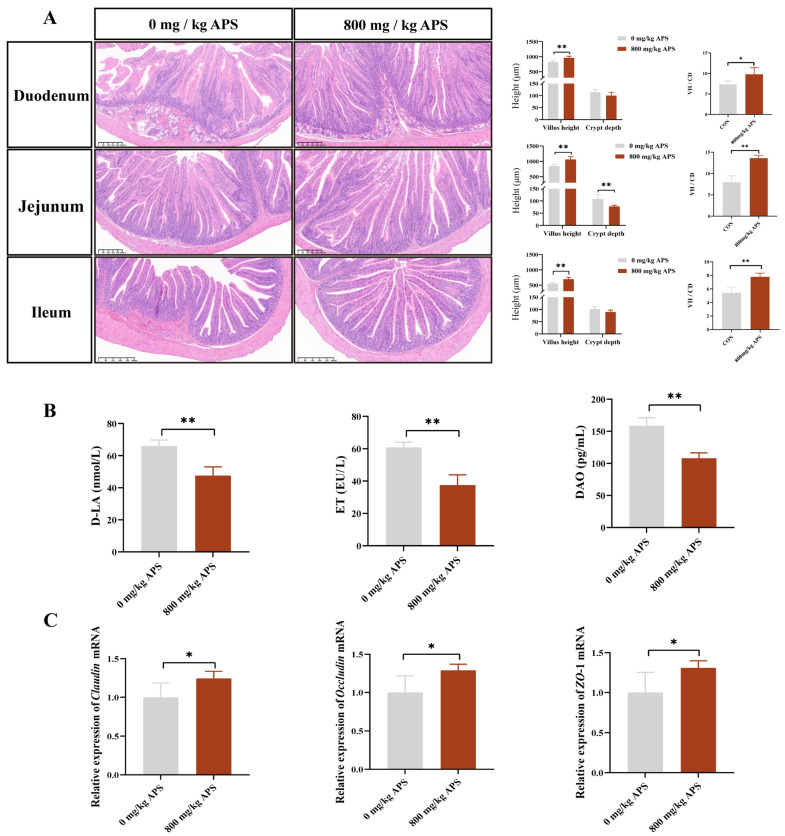
Effects of dietary supplementation with 800 mg/kg APS on intestinal barrier in rabbits. (**A**) Intestinal morphology (scale bar of duodenum and jejunum: 300 μm; scale bar of ileum: 400 μm). (**B**) Levels of intestinal permeability-related markers. (**C**) Relative expression level of tight junctions (*claudin*, *occludin*, and *ZO*-1). Data are presented as mean ± SD. The *p*-value is less than 0.05 and is identified with *. *p* < 0.01 is considered highly significant and is identified with **.

Being the organ where nutrients are digested and absorbed, the intestine is essential for animal growth [11]. Intestinal villi are an important structural basis for its function. According to the results, a significant increase in villus height was observed in the duodenum, jejunum, and ileum following APS treatment (Figure 2A). In addition, the depth of the crypts in the jejunum was significantly reduced by APS (Figure 2A). Furthermore, a significant increase in VH/CD was also observed in the duodenum, jejunum, and ileum after APS treatment (Figure 2A). The intestinal barrier can be assessed by the levels of DAO, D-LA, and Endotoxin in serum [12]. Compared with the CON, APS significantly reduced the levels of DAO, D-LA, and ET (endotoxin) in the serum (Figure 2B). At the genetic level, APS significantly upregulated the gene expression levels of *ZO*-1, *occludin*, and *claudin* of cecum (Figure 2C). The results demonstrated that APS exerts beneficial effects against intestinal barrier damage.

### 2.3. Effects of APS on Intestinal Inflammatory Factor Expression and Associated Systemic Inflammation in Rabbit

**Figure 3:**
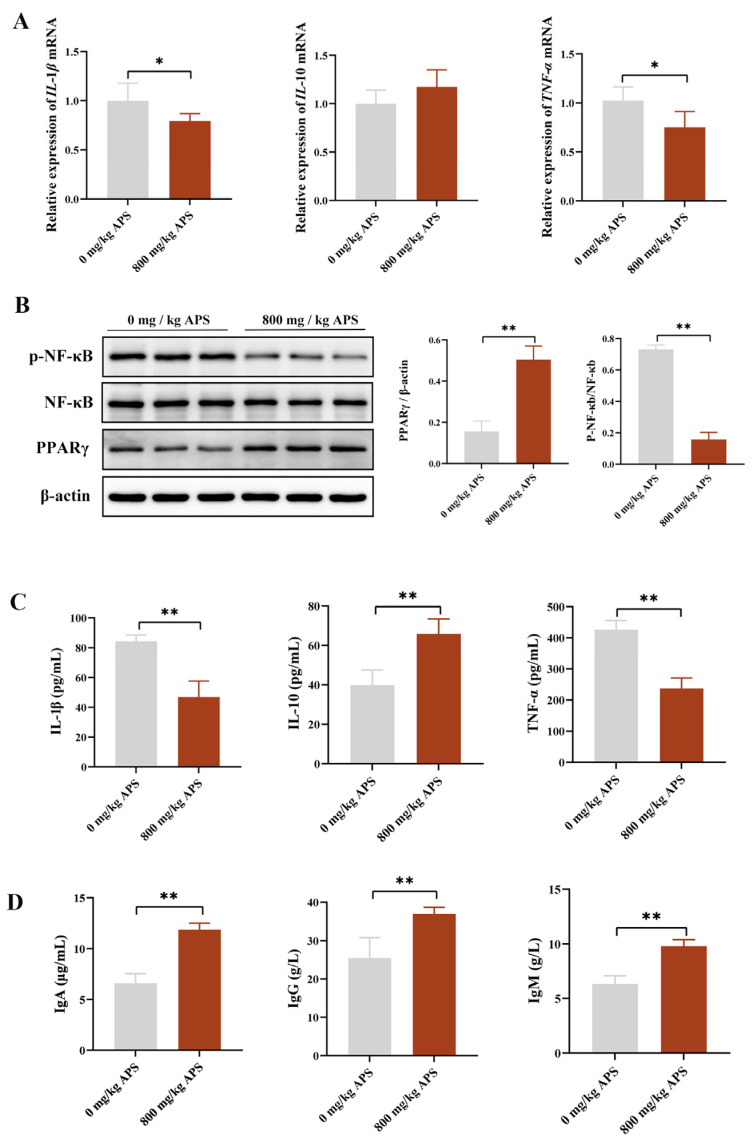
Effects of dietary supplementation with 800 mg/kg APS on intestinal inflammation in rabbits. (**A**) Relative expression of inflammatory factor (IL-1β, IL-10, and TNF-α). (**B**) Expression levels of proteins associated with the PPARγ/NF-κB pathway. (**C**) Levels of serum inflammatory cytokines. (**D**) Levels of serum immunoglobulins. Data are presented as mean ± SD. *p*-value is less than 0.05 and is identified with *. *p* < 0.01 is considered highly significant and is identified with **.

To investigate the effects of APS on the inflammatory response in rabbits, we measured the key inflammatory factors. The expression levels of the inflammatory cytokines IL-1β and TNF-α were significantly higher in the cecum of the CON (Figure 3A), which may contribute to impaired intestinal health and subsequently lead to reduced nutrient digestibility and growth performance [13]. However, APS can significantly reduce the expression of IL-1β and TNF-α (Figure 3A). WB results confirmed that APS markedly inhibited the activation of the PPARγ/NF-κB signaling pathway, as evidenced by a significant increase in PPARγ levels and a highly significant downregulation of p-NF-κB levels induced by APS (Figure 3B). Regarding systemic inflammation, serum levels of IL-1β and TNF-α showed a significant decrease (Figure 3C), while IL-10 and immunoglobulin levels (IgA, IgM, and IgG) were significantly elevated (Figure 3D). These findings suggested that APS exerted potent anti-inflammatory and immuno-enhancing effects in rabbits.

### 2.4. Effects of APS on the Microbiota in the Rabbit Cecum

**Figure 4:**
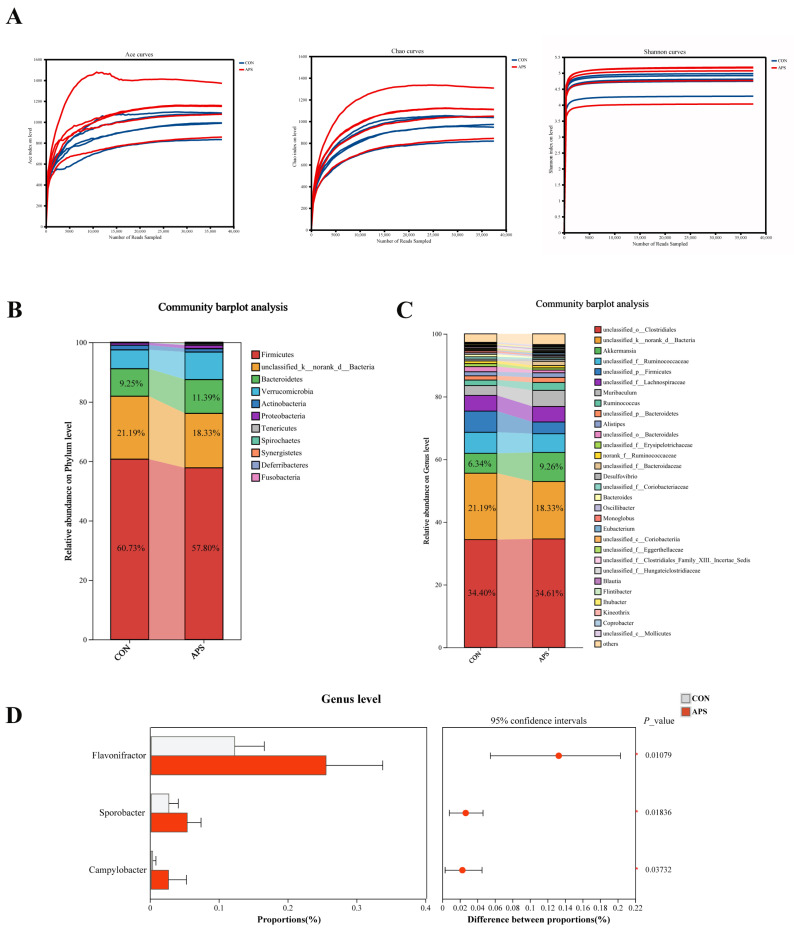
Effects of dietary supplementation with 800 mg/kg APS on cecum microbiota. (**A**) Microbial alpha diversity. (**B**) Microbial abundance at the phylum level in the cecum. (**C**) Microbial abundance at the genus level in the cecum. (**D**) Differential analysis of microbes in the cecum at the genus level. Data are presented as mean ± SD.

In rabbits, the cecum is the primary site of fermentation and harbors a diverse microbiota that aids in the digestion of indigestible substances [14]. To investigate the effects of APS on the gut microbiota, the first step was to compare the α diversity and β diversity of gut microbiota between the two groups (Figure 4A, Supplementary Figure S1). By calculating the ACE index, Chao1 index, and Shannon index, we found that APS increased microbial diversity (Figure 4A, Supplementary Table S1). Regarding the phylum level, the intestinal microbiota of both groups shared the same dominant characteristics, with Firmicutes exhibiting the highest abundance (CON: 60.73%; APS: 57.80%) (Figure 4B). At the genus level, the relative abundances of *Akkermansia* (9.26%) and *Muribaculum* (5.14%) were higher in the APS group compared to the control group (Figure 4C). Microbial differential analysis further revealed that the abundances of *Flavonifractor*, *Sporobacter*, and *Campylobacter* were significantly increased in the APS group relative to the CON group (Figure 4D).

**Figure 5:**
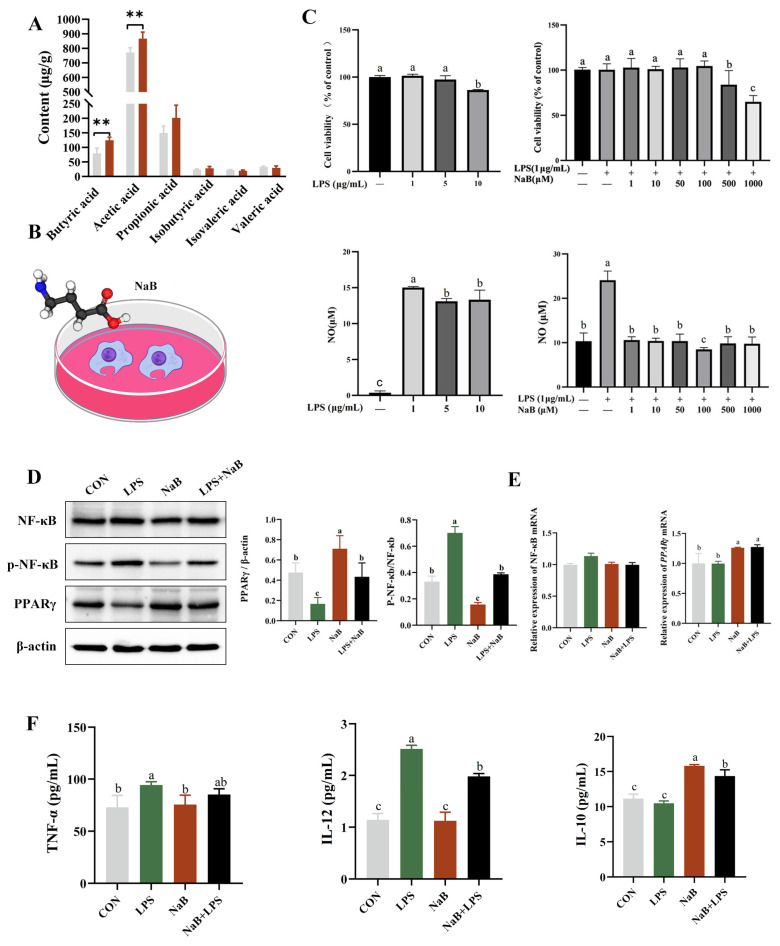
Effects of NaB on LPS-induced inflammation in the RAW264.7 model. (**A**) Content of SCFAs in the cecum of rabbits analyzed by ion chromatograph. (**B**) Schematic diagram of cell experiment. (**C**) The effects of different concentrations of NaB on the LPS-induced RAW264.7 cell inflammation model. (**D**) Changes in the levels of proteins associated with the PPARγ/NF-κB pathway in the cell (NaB: 100μM; LPS: 1 μg/mL). (**E**) Changes in the expression of genes associated with the PPARγ/NF-κB pathway in the cell (NaB: 100μM; LPS: 1 μg/mL). (**F**) Levels of inflammatory cytokines in the cell (NaB: 100μM; LPS: 1 μg/mL). Data are presented as mean ± SD. *p* < 0.01 is considered highly significant and is identified with **. Different letters indicate significant differences.

*Flavonifractor* was identified as a butyrate producer in the intestine [15]. Butyrate, a key short-chain fatty acid that maintains intestinal barrier function and suppresses inflammation, is directly related to gut health [16]. Therefore, we specifically measured butyrate levels to clarify its functional effects. APS significantly increased the level of butyric acid (*p* < 0.01) (Figure 5A). In order to study whether the anti-inflammatory effects were mediated by butyric acid, raw264.7 cells were selected, and an inflammatory model was established using LPS induction (Figure 5B). Macrophages are the most abundant mononuclear phagocytes in the intestine, which can produce pro-inflammatory cytokines, including IL-1β and TNF-α [17]. Based on the comprehensive analysis of the cell activity and NO secretion levels, this study finally confirmed that the intervention dose of LPS was 1 μg/mL, and that of NaB was 100 μM (Figure 5C). NF-κB is a key transcription factor whose activity can be activated by LPS [18]. PPARγ is an upstream regulatory molecule of the NF-κB signaling pathway and exerts significant inflammation-suppressing biological properties by inhibiting the cascade reaction of the NF-κB signaling pathway [19]. The results of this study showed that LPS treatment significantly upregulated p-NF-κB levels while significantly downregulating PPAR-γ expression (*p* < 0.05). NaB intervention can effectively alleviate the above-mentioned molecular expression abnormalities induced by LPS (Figure 5D,E) (*p* < 0.05). To evaluate the inhibitory effect of sodium butyrate on LPS-induced cytokine production, cytokine levels were assessed by ELISA. As shown in Figure 5F, LPS-induced upregulation of IL-12 and downregulation of IL-10 were inhibited by butyrate in RAW 264.7 cells (Figure 5F) (*p* < 0.05).

## 3. Discussion

Supplementing pharmacologically effective feed additives to weaning diets enhances the immune function of animals, representing an effective strategy to lower disease susceptibility and enhance pathogen resistance in weaned rabbits. APS, with its anti-inflammatory, antioxidant, and immune-enhancing properties, is an excellent feed additive. In this experiment, compared with the CON group, adding 800 mg/kg APS to the diet significantly increased the ADG and reduced the F/G ratio in rabbits, indicating that dietary APS supplementation can exert beneficial effects on growth performance. Slaughter performance and meat quality are important indicators for evaluating the production level of meat rabbits [20]. In this study, increasing the APS supplementation dose was associated with higher eviscerated and semi-eviscerated weights, as well as increased eviscerated and semi-eviscerated yield percentages. Drip loss in muscle was significantly reduced by APS, indicating that APS can improve slaughter performance and water-holding capacity in rabbits. Currently, there is limited research on the effects of alfalfa polysaccharides on meat quality. However, alfalfa polysaccharides have been demonstrated to possess immunomodulatory activity and can improve intestinal morphology [10]. Therefore, we hypothesize that supplementing APS in feed may improve slaughter performance and water-holding capacity by enhancing nutrient utilization in the diet through improving gut health in rabbits. Notably, rabbits supplemented with 800 mg/kg APS in this study were found to have the lowest F/G ratio and drip loss, providing indirect support for our hypothesis.

To validate the above hypothesis, we subsequently evaluated the effects of APS on intestinal morphology and barrier function in rabbits. The results of this study demonstrated that APS increased villus height in the duodenum and jejunum while decreasing crypt height in the jejunum. The results of Xie’s research also support our findings, demonstrating that APS significantly improves intestinal morphology in the duodenum and jejunum of mice, providing preliminary evidence of its beneficial effects on gut health [10]. Secondly, barrier function in the gut was significantly improved by APS, as evidenced by increased expression of intestinal junction proteins and decreased serum levels of D-LA, ET, and DAO. Maintaining gut barrier integrity is crucial for both intestinal and systemic health, and impaired barrier function leads to forced activation of immune cells and chronic tissue inflammation [21]. In this study, the intestinal barrier was effectively enhanced by APS, which further alleviated intestinal inflammation. Expression of IL-1β and TNF-α in the cecum was reduced by APS treatment, and activity of the PPARγ/NF-κB signaling pathway was downregulated, demonstrating the anti-inflammatory effects of APS. Systemically, APS reduced serum levels of IL-1β and TNF-α and increased the contents of IgA, IgG, IgM, and IL-10, further confirming its ability to enhance the body’s immune homeostasis. In summary, dietary APS supplementation in weaned rabbits was associated with enhanced intestinal health, as indicated by improved intestinal morphology, strengthened barrier function, and suppressed inflammatory responses both locally and systemically. Collectively, these improvements in intestinal homeostasis may contribute to the previously observed enhancements in growth performance and meat quality. We propose that this could occur through a dual mechanism: optimized intestinal function likely supports more efficient nutrient utilization, while a reduced inflammatory burden may spare energy and nutrients, potentially redirecting them toward growth and muscle development. How does APS regulate gut health? Research indicates that polysaccharides, as one of the abundant dietary components in the gut microbiota, can exert multiple regulatory functions by participating in the gut microbiome–host symbiotic system [22]. Previous investigation has demonstrated that *Astragalus polysaccharides* increased butyrate levels by modulating the gut microbiota in mice. Butyrate could reduce oxidative stress and inflammatory responses in the brain by crossing the blood–brain barrier [23]. Similarly, *Atractylodes macrocephala Koidz polysaccharide* ameliorated the imbalance of the gut microbiota in colitis mice and elevated levels of tryptophan metabolites, thereby suppressing intestinal inflammation and maintaining intestinal barrier integrity [24]. Based on the previous studies, we speculate that alfalfa polysaccharides may also work in a microbiota-dependent manner. The results indicated that APS treatment significantly increased the abundance of *Flavonifractor*, *Sporobacter*, and *Campylobacter* in the cecum. As previously mentioned, a key functional attribute of *Flavonifractor* is its ability to generate butyrate. In line with this, the concentration of butyrate in the cecal contents also showed a significant increase. While butyrate is the preferred energy source for gut epithelial cells, it also functions as a key signaling molecule regulating the host immune response [21,25]. The findings of this study suggest that the regulatory role of APS in gut health may involve a pathway that enriches *Flavonifractor* to increase butyrate levels, which may, in turn, contribute to improving gut microbiota and barrier function.

To validate the immunomodulatory effects of butyric acid, we conducted in vitro experiments using Raw264.7 cells. LPS can induce macrophage polarization toward the M1 phenotype and stimulate the release of pro-inflammatory mediators, including IL-1β, TNF-α, and IL-12 [26,27,28]. NO is a key effector molecule of M1 macrophages that suppresses cell proliferation and induces oxidative stress [29]. Our results indicated that butyrate significantly inhibited LPS-stimulated NO production in macrophages. NO is a downstream product of iNOS, whose expression is modulated by the NF-κB signaling pathway [30]. Notably, during this process, the level of the anti-inflammatory cytokine IL-10 was not significantly induced by LPS. This observation aligns with the characterization of LPS as a primary driver of the classical pro-inflammatory response, further confirming that the inflammatory model established in this study exhibits a distinct M1-polarized tendency [31]. Our data showed that butyrate treatment was associated with reduced activity of the PPARγ/NF-κB pathway and lower levels of LPS-induced IL-12. These results suggest that the anti-inflammatory effect of butyrate may involve, at least in part, the modulation of the NF-κB signaling pathway.

## 4. Materials and Methods

### 4.1. Animals and Diets

In the experiment, 42-day-old weaned *Hyplus rabbits* in good health and of similar body weights were selected. After an acclimatization period, rabbits were randomly assigned to the four experimental groups based on body weight using a random number table, ensuring no significant difference in initial body weight across groups. The four experimental groups were the control group (basal diet), the 400 mg/kg APS group (basal diet supplemented with APS at 400 mg/kg), the 800 mg/kg APS group (basal diet supplemented with APS at 800 mg/kg), and the 1200 mg/kg APS group (basal diet supplemented with APS at 1200 mg/kg). Each treatment comprised five replicates, with four rabbits per replicate. The trial was conducted for a period of 30 days following a 5-day adaptation period. The experimental diet was designed according to the meat rabbit feeding standards outlined in “Nutrition of Domestic Rabbits”, with feed composition as shown in Table 2. In this study, APS (purity ≥ 80%) was purchased from Kang’ermei Biotechnology Co., Ltd. (Xi’an, China).

### 4.2. Growth Performance

At the start (initial weight) and end (terminal weight) of the trial, fasting weights were recorded for each group of rabbits to calculate average daily gain (ADG). Daily feed intake was measured by providing a known amount of feed each morning and weighing the leftovers before the next feeding, with average daily feed intake (ADFI) = (feed provided − leftover weight)/number of rabbits per cage/number of days. Feed conversion ratio (FCR) was calculated as FCR = ADG/ADFI.

### 4.3. Slaughter Performance

At the end of the experiment, five meat rabbits were selected from the control group to determine the optimal application effects of APS for slaughter. The five meat rabbits had body weights close to the group average. Slaughter, eviscerated, and semi-eviscerated weights were determined. The following formulas were applied to calculate dressing percentages: eviscerated yield (%) = (eviscerated weight/slaughter weight) × 100%; semi-eviscerated yield (%) = (semi-eviscerated weight/slaughter weight) × 100%.

### 4.4. Meat Quality

After slaughter, the *Longissimus dorsi* muscle was collected for meat quality assessment. The pH of the muscle was measured at 45 min post-slaughter using a testo 205 pH meter. Meat color parameters—lightness (L*), redness (a*), and yellowness (b*)—were determined using a CR-10 colorimeter (KONICA MINOLTA, Tokyo, Japan).

A sample of the *Longissimus dorsi* was weighed (m_1_, g), placed into an EZ test tube, and stored in a refrigerator at 4 °C. After 24 h, the sample was reweighed (m_2_, g), with drip loss (%) = [(m_1_ − m_2_)/m_1_] × 100%.

Muscle samples were cut along the muscle fiber direction into strips measuring 4–6 cm in length with a cross-sectional area of 1 cm^2^ (1 × 1 cm). Shear force was measured by a texture analyzer and expressed in Newtons (N).

### 4.5. Serum Collection

The rabbit with the body weight closest to the mean body weight of each treatment group was selected from each replicate, with one rabbit per replicate and a total of five rabbits per group. Subsequently, blood was collected via auricular venipuncture and centrifuged at 3000 r/min for 10 min. After centrifugation, serum was aspirated and aliquoted into 1.5 mL centrifuge tubes for storage.

### 4.6. Intestinal Morphology

Intestinal tissue was slowly rinsed with PBS and then stored in 4% formaldehyde solution. The tissue, having been dehydrated and cleared, was embedded in paraffin blocks and then sectioned. Sections were stained with hematoxylin and eosin. Villus height (VH) and crypt depth (CD) were measured using a microscope, and the villus height-to-crypt depth ratio (VH/CD) was calculated.

### 4.7. Enzyme-Linked Immunosorbent Assay (ELISA)

Serological assays were performed strictly in accordance with the reagent manual. ELISA kits for IL-1β (rabbit: YJ027836), TNF-α (rabbit: YJ027766; mice: YJ037868), IgA (rabbit: YJ027971), IgG (rabbit: YJ027242), IgM (rabbit: YJ027243), DAO (rabbit: YJ027888), D-LA (rabbit: YJ521396), endotoxin (rabbit: YJ522961), IL-10 (rabbit: YJ027828; mice: YJ037873), IL-12 (mice: YJ037868), and NO (ml001883) were sourced from Enzyme-linked Biotechnology (Shanghai, China).

### 4.8. 16s rRNA

Cecum-derived genomic DNA, extracted with the FastPure Stool DNA isolation kit (Shanghai Meiji Yuhua Bio-Pharmaceutical Technology Co., Ltd., Shanghai, China) following the provided guidelines, served directly as templates for subsequent PCR amplification. The amplified products were recovered, purified, and used to construct a library. Sequencing was performed on the MiSeq PE300 or NovaSeq PE250 platform (Majorbio Bio-Pharm Technology Co., Ltd., Shanghai, China). Data analysis was conducted on the Majorbio Cloud Platform (https://www.majorbio.com/). UPARSE software (http://drive5.com/uparse/, version 7.1) was used to perform OTU clustering on sequences based on a 97% similarity threshold. The RDP classifier was utilized to conduct species taxonomic annotation for each sequence and aligned with the Silva 16S rRNA database (version 138).

### 4.9. Detection of SCFAs

The contents of the cecum (0.5 g) were diluted with deionized water (a mass-to-volume ratio of 1:19). The diluted sample was sonicated for 30 min, followed by centrifugation at 10,000 rpm for 15 min. The resulting supernatant was then subjected to a second round of centrifugation under the same conditions. An equal volume of supernatant was transferred onto a 0.22 μm microporous membrane filter for volatile fatty acid concentration determination using an ion chromatograph (Thermo Scientific Dionex ICS-5000+SP, Thermo Scientific, Waltham, MA, USA).

### 4.10. Cell Culture

The RAW264.7 macrophage cell line was cultured using DMEM supplemented with 10% fetal bovine serum (FBS). Cells were passaged when reaching approximately 80–90% confluency. Cells were divided into four experimental groups: control (CON), LPS (lipopolysaccharide, O55:B5, Sigma, L2880), NaB (sodium butyrate, Sigma, V900464), and LPS + NaB. For subsequent experiments, cells were seeded at a density of 1 × 10^4^ cells per well. Cell viability testing was performed according to the kit instructions (Beyotime, Wuhan, China). In brief, after cell seeding and attachment, diluted LPS (0, 1.0, 5.0, and 10 μg/mL) or NaB (0, 1, 10, 50, 100, 500, and 1000 μM) was added to the cells. Each treatment was performed in triplicate, followed by incubation at 37 °C. For cell viability assessment, the original medium was removed, and cells were washed twice with PBS. A working solution was prepared by mixing DMEM and CCK-8 reagent at a 10:1 ratio, and 100 μL was added per well. After incubation at 37 °C for 2 h, the absorbance was measured at 450 nm using a microplate reader (Thermo Scientific, Waltham, MA, USA).

### 4.11. Western Blot

The operation was carried out in accordance with the standard protocol for Western blotting [32]. In brief, protein extraction was performed by lysing cells on ice with RIPA buffer containing protease and phosphatase inhibitors, followed by centrifugation of lysates at 12,000 rpm for 15 min at 4 °C and supernatant collection; protein concentration was quantified via a BCA assay kit (Beyotime, Wuhan, China) with β-actin as the loading control for normalization; and equal amounts of protein (20–30 μg) were separated by SDS-PAGE and wet-transferred onto PVDF membranes, which were then blocked with 5% skimmed milk for 1 h at room temperature, incubated with antibodies, and visualized using ECL substrate, with signals captured by a chemiluminescence imaging system and analyzed via ImageJ software 1.43 (National Institutes of Health, Bethesda, MD, USA). For rabbit tissue experiments, the primary antibodies were β-actin (BIOSS, bs-0061R, 1:5000), NF-κB p65 (ZNGBIO, 380172, 1:1000), p-NF-κB p65 (CST, 3033, 1:1000), and PPARγ (BIOSS, bs-0530R, 1:2000). For RAW264.7 cell experiments, the primary antibodies were β-actin (same as above), NF-κB p65 (ZNGBIO, 823310, 1:1000), p-NF-κB p65 (Abmayt, TP56372F, 1:2000), and PPARγ (CST, 2443S, 1:1000). The secondary antibody for both were HRP-Goat Anti-Rabbit (Scracare, 5220-0336, 1:50,000) and HRP-Goat Anti-Mouse (Scracare, 5220-0341, 1:50,000).

### 4.12. qPCR

Total RNA was extracted from the cecum and cell using Trizol reagent (Vazyme, Nanjing, China). First-strand cDNA was synthesized using the HiScript III 1st Strand cDNA Synthesis Kit (Vazyme, Nanjing, China). qPCR was performed using ChamQ Universal SYBR qPCR Master Mix (Vazyme, Nanjing, China) according to the manufacturer’s instructions. Relative gene expression was calculated using the 2^−ΔΔct^ method. Primer sequences are listed in Table 3.

### 4.13. Statistical Analysis

The experimental data were presented as mean ± SD, and statistical analysis was conducted using IBM SPSS Statistics 22.0 (IBM, New York, NY, USA). For the data from animal-based experiments, one-way analysis of variance (ANOVA) followed by Tukey’s honestly significant difference (HSD) post hoc test was used to analyze differences among groups; an independent samples *t*-test was used for comparisons between two groups (con vs. 800 mg/kg APS). For the data from cell-based experiments, one-way analysis of variance (ANOVA) followed by Tukey’s honestly significant difference (HSD) post hoc test was used. The differences between treatments are considered statistically significant when the *p*-value is less than 0.05 and is identified with *. *p* < 0.01 is considered highly significant and is identified with **. Data visualization was performed using GraphPad Prism 8.0.

## 5. Conclusions and Outlook

In summary, our results indicate that APS supplementation is associated with an increased abundance of the butyrate-producing bacterium *Flavonifractor* and a concomitant elevation in cecal butyrate concentration. This suggests that the anti-inflammatory effects observed in vivo may be linked, at least in part, to butyrate-mediated modulation of inflammatory pathways, PPARγ/NF-κB, as supported by our in vitro findings in macrophages. However, it is important to note the limitations of the current mechanistic exploration. Firstly, certain in-depth post-slaughter parameters were assessed only between the control and the high-dose APS group, which limits our ability to construct a comprehensive dose–response curve and to extrapolate the findings to other supplementation levels; future dose-ranging studies should include all experimental groups. Secondly, the use of RAW264.7 macrophages, which may not fully recapitulate the intestinal immune micro-environment. Thirdly, the relatively modest sample size may limit the detection of more subtle effects. The enrichment of *Flavonifractor* likely represents a key, but not exclusive, event within a broader microbial network shift induced by APS. Future studies employing fecal microbiota transplantation, germ-free models, or specific pathway antagonists are warranted to establish causal relationships and elucidate the precise mechanistic hierarchy.

## Figures and Tables

**Table 1:** Effect of diets when adding different levels of APS on slaughter performance and meat quality of rabbits (47 days–77days).

Items		APS Level (mg/kg)				
	CON	400.00	800.00	1200.00	SEM	*p*-Value
Slaughter weight, g	2256.00 ^b^	2310.11 ^ab^	2430.00 ^ab^	2482.00 ^a^	32.56	0.035
Eviscerated weight	1188.00 ^ab^	1158.00 ^b^	1248.00 ^ab^	1302.00 ^a^	18.97	0.020
Semi-eviscerated weight	1267.32 ^ab^	1239.52 ^b^	1340.43 ^ab^	1396.40 ^a^	20.58	0.015
Eviscerated yield percentage	52.73	50.11	51.35	52.45	0.39	0.050
Semi-eviscerated yield percentage	56.23 ^ab^	53.64 ^b^	55.16 ^ab^	56.25 ^a^	0.38	0.035
pH45min	6.51	6.63	6.91	6.93	0.08	0.119
L*	58.89	56.07	55.68	58.94	0.61	0.083
a*	6.21	7.95	6.84	6.84	0.37	0.490
b*	3.70	4.63	4.89	4.35	0.25	0.403
Drip loss/%	2.50 ^a^	1.49 ^b^	1.47 ^b^	1.69 ^b^	0.13	0.006
Shear force/N	24.11	23.94	22.69	23.10	0.56	0.807

^a, b^ Within a row, different superscripts indicate significant differences at the *p* < 0.05 level. The same is applicable to the table below. L*, lightness; a*, redness; b*, yellowness.

**Table 2:** Basic diet composition and nutritional level.

Ingredients	%	Nutrient Levels	
Corn	31.67	Digestive energy (MJ/kg)	10.23
Wheat bran	19.33	Crude protein (%)	15.44
Soybean meal	17.00	Crude fiber (%)	14.61
Wheat middlings	5.12	Neutral detergent fiber (%)	28.91
Peanut shells	22.88	Acid detergent fiber (%)	17.95
Premix ^1^	4.00	Crude fat (%)	2.70
Total	100.00	Ca (%)	0.60
		P (%)	0.40

^1^ The premix provides the following per kg of basal diet: Fe, 70 mg per kg of full price; Cu, 20 mg; Zn, 70 mg; Mn, 10 mg; Co, 0.15 mg; I, 0.2 mg; Se, 0.20 mg; VA, 10,000 IU; VD, 900 IU; VE, 50 mg; VK, 2 mg; thiamin, 2 mg; riboflavin, 6 mg; pantothenic acid, 50 mg; pyridoxine, 2 mg; VB12, 0.02 mg; nicotinic acid, 50 mg; choline, 1000 mg; biotin, 0.2 mg.

**Table 3:** The sequences of the primers.

Species	Gene	Sequence (5′—3′)
*Mice*	*PPARγ*	F: GAAGGAGAAGCTGTTGGCGGAG
		R: GCGGGAAGGACTTTATGTATGA
	*NF-κB*	F: GGAAGCAAATCTATACCCCCAT
		R: ACAGCCCTCAGAATCCACCGTG
	*β-actin* (reference gene)	F: GTGCGTGACATCAAAGAGAA
		R: GAAGGAAGGCTGGAAAAGAG
*Rabbit*	*TNF-α*	F: AGCCCACGTAGTAGCAAACC
		R: TGAGTGAGGAGCACGTAGGA
	*IL-1β*	F: TCTGCAACACCTGGGATGAC
		R: TCAGCTCATACGTGCCAGAC
	*IL-10*	F: TCACCGATTTCTCCCCTGTG
		R: ATGTCAAACTCACTCATGGCTT
	*Occludin*	F: CCGTATCCAGAGTCCTACAAGT
		R: GTCCGTCTCGTAGTGGTCTT
	*ZO-1*	F: CCGCTCATACCTTCCTCTCA
		R: GTCATTCACCTCCTTCTTGTTCTC
	*Claudin*	F: ACAGCATGGTATGGCAACAG
		R: CGAGGACAAGAACAGCAAAGT
	*GAPDH* (reference gene)	F: TGTTTGTGATGGGCGTGAA
		R: CCTCCACAATGCCGAAGT

## Data Availability

The original contributions presented in this study are included in the article/supplementary material. Further inquiries can be directed to the corresponding author(s). Microbial data can be obtained at PRJNA1398611.

## Supplementary Materials

The following supporting information can be downloaded at https://www.mdpi.com/article/10.3390/ijms27020994/s1.

## Abbreviations

Alfalfa polysaccharide (APS); average daily gain (ADG); average daily feed intake (ADFI); feed conversion ratio (FCR); villus height (VH); crypt depth (CD); lipopolysaccharide (LPS); sodium butyrate (NaB); tumor necrosis factor (TNF); nuclear factor kappa-B (NF-κB); peroxisome proliferator-activated receptor γ (PPARγ).

## References

[1] Mancini S., Paci G. (2021). Probiotics in Rabbit Farming: Growth Performance, Health Status, and Meat Quality. Animals.

[2] Marlier D., Dewrée R., Lassence C., Licois D., Mainil J., Coudert P., Meulemans L., Ducatelle R., Vindevogel H. (2006). Infectious Agents Associated with Epizootic Rabbit Enteropathy: Isolation and Attempts to Reproduce the Syndrome. Vet. J..

[3] Wang Y., Zhang Y., Ren H., Fan Z., Yang X., Zhang C., Jiang Y. (2023). Dietary yucca extract and Clostridium butyricum promote growth performance of weaned rabbits by improving nutrient digestibility, intestinal development, and microbial composition. Front. Vet. Sci..

[4] Wang Y., Jia X., Guo Z., Li L., Liu T., Zhang P., Liu H. (2022). Effect of Dietary Soybean Saponin Bb on the Growth Performance, Intestinal Nutrient Absorption, Morphology, Microbiota, and Immune Response in Juvenile Chinese Soft-Shelled Turtle (Pelodiscus Sinensis). Front. Immunol..

[5] Yang C., Wang S., Qi Y., Jin Y., Guan R., Huang Z. (2025). Enhancing Growth Performance in Liangshan Black Sheep through Fermented Onion: Insights from Transcriptomics and Metabolomics. Front. Vet. Sci..

[6] Zhao R.-H., Yang F.-X., Bai Y.-C., Zhao J.-Y., Hu M., Zhang X.-Y., Dou T.-F., Jia J.-J. (2023). Research Progress on the Mechanisms Underlying Poultry Immune Regulation by Plant Polysaccharides. Front. Vet. Sci..

[7] Chi Y., Li Y., Zhang G., Gao Y., Ye H., Gao J., Wang P. (2018). Effect of Extraction Techniques on Properties of Polysaccharides from Enteromorpha Prolifera and Their Applicability in Iron Chelation. Carbohydr. Polym..

[8] Yu X., Mu N., Liu X., Shang Y., Wang D., Li F. (2023). A Green Method for Decolorization of Polysaccharides from Alfalfa by S-8 Macroporous Resin and Their Characterization and Antioxidant Activity. RSC Adv..

[9] Li Z., Sang R., Feng G., Feng Y., Zhang R., Yan X. (2024). Microbiological and Metabolic Pathways Analysing the Mechanisms of Alfalfa Polysaccharide and Sulfated Alfalfa Polysaccharide in Alleviating Obesity. Int. J. Biol. Macromol..

[10] Xie Y., Wang L., Sun H., Wang Y., Yang Z., Zhang G., Yang W. (2019). Immunomodulatory, Antioxidant and Intestinal Morphology-Regulating Activities of Alfalfa Polysaccharides in Mice. Int. J. Biol. Macromol..

[11] Li R., Li X., Huang T., Wang Y., Xue M., Sun S., Yan D., Song G., Sun G., Li M. (2020). Influence of cecotrophy on fat metabolism mediated by caecal microorganisms in New Zealand white rabbits. J. Anim. Physiol. Anim. Nutr..

[12] Li L., Wei X.-F., Yang Z.-Y., Zhu R., Li D.-L., Shang G.-J., Wang H.-T., Meng S.-T., Wang Y.-T., Liu S.-Y. (2023). Alleviative Effect of Poly-β-Hydroxybutyrate on Lipopolysaccharide-Induced Oxidative Stress, Inflammation and Cell Apoptosis in Cyprinus Carpio. Int. J. Biol. Macromol..

[13] Chen X., Li Z., Pu J., Cai J., Zhao H., Jia G., Liu G., Tian G. (2023). Dietary Betaine Improves the Intestinal Health and Growth Performance of Heat-Stressed Growing Rabbits in Summer. J. Anim. Sci..

[14] Liu B., Cui Y., Ali Q., Zhu X., Li D., Ma S., Wang Z., Wang C., Shi Y. (2022). Gut Microbiota Modulate Rabbit Meat Quality in Response to Dietary Fiber. Front. Nutr..

[15] Vital M., Karch A., Pieper D.H. (2017). Colonic Butyrate-Producing Communities in Humans: An Overview Using Omics Data. mSystems.

[16] Stoeva M.K., Garcia-So J., Justice N., Myers J., Tyagi S., Nemchek M., McMurdie P.J., Kolterman O., Eid J. (2021). Butyrate-Producing Human Gut Symbiont, Clostridium Butyricum, and Its Role in Health and Disease. Gut Microbes..

[17] Zhang K., Guo J., Yan W., Xu L. (2023). Macrophage Polarization in Inflammatory Bowel Disease. Cell Commun. Signal..

[18] Wei H., Yu C., Zhang C., Ren Y., Guo L., Wang T., Chen F., Li Y., Zhang X., Wang H. (2023). Butyrate Ameliorates Chronic Alcoholic Central Nervous Damage by Suppressing Microglia-Mediated Neuroinflammation and Modulating the Microbiome-Gut-Brain Axis. Biomed. Pharmacother..

[19] Cao S., Lv B., Tai Y., Zuo H.X., Xing Y., Surh Y.-J., Li M.Y., Ma J., Jin X. (2025). Formononetin Ameliorates DSS-Induced Colitis by Inhibiting the MAPK/PPAR-γ/NF-κB/ROS Signaling Pathways. Toxicol. Appl. Pharmacol..

[20] Wang Z., Xing T., Zhang L., Zhao L., Gao F. (2024). Effects of Substituting Soybean Meal with Fermented Rapeseed Meal Mixture on the Growth Performance, Slaughter Performance, Meat Quality, Blood Biochemical Indices and Intestinal Barrier Function in Langshan Chickens. Poult. Sci..

[21] Liu L., Chen T., Xie Z., Zhang Y., He C., Huang Y. (2024). Butyric Acid Alleviates LPS-Induced Intestinal Mucosal Barrier Damage by Inhibiting the RhoA/ROCK2/MLCK Signaling Pathway in Caco2 Cells. PLoS ONE.

[22] Qiao Y., Liu C., Guo Y., Zhang W., Guo W., Oleksandr K., Wang Z. (2022). Polysaccharides derived from Astragalus membranaceus and Glycyrrhiza uralensis improve growth performance of broilers by enhancing intestinal health and modulating gut microbiota. Poult. Sci..

[23] Wei X., Xin J., Chen W., Wang J., Lv Y., Wei Y., Li Z., Ding Q., Shen Y., Xu X. (2023). Astragalus Polysaccharide Ameliorated Complex Factor-Induced Chronic Fatigue Syndrome by Modulating the Gut Microbiota and Metabolites in Mice. Biomed. Pharmacother..

[24] Zhang Q.-W., Yang M.-J., Liao C.-Y., Taha R., Li Q.-Y., Abdelmotalab M.I., Zhao S.-Y., Xu Y., Jiang Z.-Z., Chu C.-H. (2025). Atractylodes Macrocephala Koidz Polysaccharide Ameliorates DSS-Induced Colitis in Mice by Regulating the Gut Microbiota and Tryptophan Metabolism. Br. J. Pharmacol..

[25] Manrique Vergara D., González Sánchez M.E. (2017). Short chain fatty acids (butyric acid) and intestinal diseases. Nutr. Hosp..

[26] Benoit M., Desnues B., Mege J.-L. (2008). Macrophage Polarization in Bacterial Infections. J. Immunol..

[27] Locati M., Curtale G., Mantovani A. (2020). Diversity, Mechanisms, and Significance of Macrophage Plasticity. Annu. Rev. Pathol..

[28] Rhee I. (2016). Diverse Macrophages Polarization in Tumor Microenvironment. Arch. Pharm. Res..

[29] Kashfi K., Kannikal J., Nath N. (2021). Macrophage Reprogramming and Cancer Therapeutics: Role of iNOS-Derived NO. Cells.

[30] Shi Y., Liu H., Liu H., Yu Y., Zhang J., Li Y., Luo G., Zhang X., Xu N. (2020). Increased Expression Levels of Inflammatory Cytokines and Adhesion Molecules in Lipopolysaccharide-induced Acute Inflammatory apoM−/− Mice. Mol. Med. Rep..

[31] Huang X., Li Y., Fu M., Xin H. (2018). Polarizing Macrophages In Vitro. Methods Mol. Biol..

[32] Mahmood T., Yang P.-C. (2012). Western Blot: Technique, Theory, and Trouble Shooting. N. Am. J. Med. Sci..

