# Supplementary material for: Alfalfa Polysaccharide Improves Rabbit Growth by Modulating Gut Microbiota and Suppressing Inflammation Through PPARγ/NF-κB Pathway
Source: Int J Mol Sci. 2026 Jan 19;27(2):994. doi: 10.3390/ijms27020994 (PMC12842300; doi:10.3390/ijms27020994)
Supplement: Supplementary file 1 [file ijms-27-00994-s001.zip › Supplementary Table S1.pdf]

Table S1 α diversity analysis of gut microbiota

	CON	APS
ACE	992.84±110.11	1118.96±180.77
Chao1	965.64±100.57	1080.01±162.99
Shannon	4.75±0.28	4.85±0.48
