# Supplementary figures and images for: Alfalfa Polysaccharide Improves Rabbit Growth by Modulating Gut Microbiota and Suppressing Inflammation Through PPARγ/NF-κB Pathway
Source: Int J Mol Sci. 2026 Jan 19;27(2):994. doi: 10.3390/ijms27020994 (PMC12842300; doi:10.3390/ijms27020994)

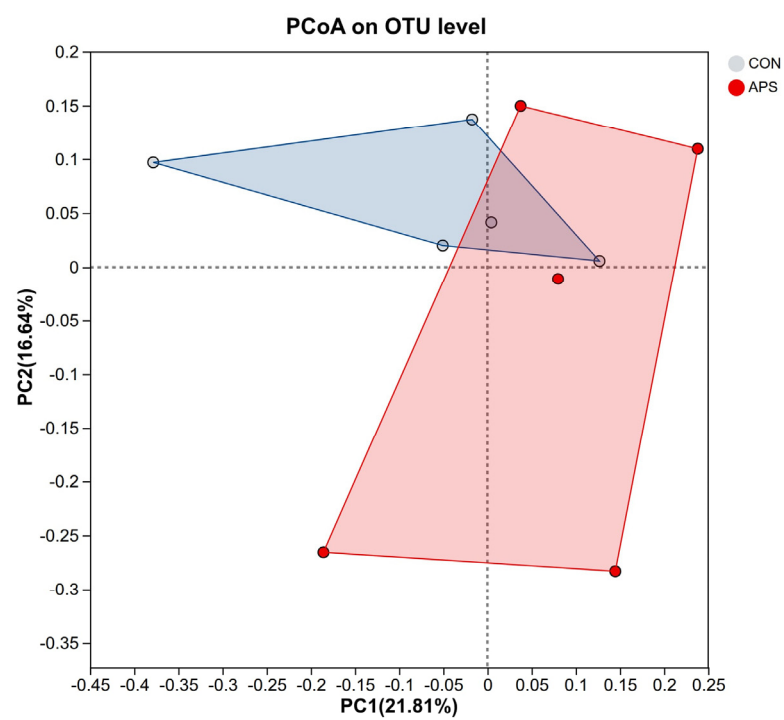

Figure S1: Principal co-ordinates analysis (PCoA).

Supplement: Supplementary file 1 [file ijms-27-00994-s001.zip › Supplementary Figure S1.pdf]
